# Chief’s seminar: turning interns into clinicians

**DOI:** 10.12688/f1000research.5221.1

**Published:** 2014-09-08

**Authors:** Christopher Dittus, Vanya Grover, Georgia Panagopoulos, Kenar Jhaveri

**Affiliations:** 1 Department of Internal Medicine, Lenox Hill Hospital, North Shore-LIJ Health System, New York, 10075, USA; 2 Department of Research, Lenox Hill Hospital, North Shore-LIJ Health System, New York, 10075, USA; 3 Department of Internal Medicine, North Shore-LIJ Health System and Hofstra North Shore LIJ School of Medicine, Great Neck, 11021, USA

## Abstract

**Background**: Recent changes in healthcare delivery have necessitated residency education reform. To adapt to these changes, graduate medical education can adopt a chief resident-led clinical curriculum. Chief residents are ideal clinical instructors, as they are recent graduates who have excelled in their residency programs. To effectively use the limited time available for education, chief residents can implement active learning techniques. We present a chief resident-led, small-group, problem-based curriculum for teaching first-year internal medicine residents, and provide preliminary data supporting the efficacy of this approach.

**Methods**: The seminar consisted of 11 4-week modules. Week 1 was a team-based crossword competition. Weeks 2-4 were small-group, problem-based clinical reasoning sessions taught by chief residents. The program was evaluated via pre- and post-module multiple-choice tests. Resident satisfaction data were collected via self-reported, anonymous surveys.

**Results**: Preliminary results revealed a statistically significant increase from pre-test to post-test score for 9 of the 11 modules. The chest pain, fever, abdominal pain, shock, syncope, jaundice, dizziness, anemia, and acute kidney injury modules achieved statistical significance. Additionally, resident satisfaction surveys show that this teaching approach was an enjoyable experience for our residents.

**Discussion**: Our chief seminar is an evidence-based, clinical reasoning approach for graduate medical education that uses active learning techniques. This is an effective and enjoyable method for educating internal medicine residents. Because of its reproducibility, it can be applied throughout residency education.

## Background

The changing landscape of healthcare delivery has made residency education reform a necessity ^1^. In the hospital, residents must navigate an increasingly complicated healthcare system, while having to expeditiously diagnose, treat, and discharge patients. Furthermore, medical knowledge is increasing in both the basic and clinical sciences. This is evidenced by the dramatic rise in the quantity of publications in the medical literature, particularly randomized controlled trials ^2^. To keep pace with these demands, graduate medical education can adopt a chief resident-led clinical curriculum. Chief residents are ideal clinical instructors, as they have the knowledge base of an attending while still understanding the learning needs of residents. To effectively use the limited time available for education, chief residents can implement active learning techniques. Approaches that fall under active learning are: problem-based learning ^3– 6^; collaborative group work; and peer instruction ^7, 8^. Small group learning is an effective way to apply these techniques ^9, 10^. The application of active learning principles to medical education has been increasingly promoted in the medical literature ^1, 11– 13^. Active learning also allows for the addition of creative modalities, such as concept mapping, games, puzzles, and extrinsic rewards ^4, 9, 14, 15^. We present a chief resident-led, small-group, problem-based curriculum for teaching first-year internal medicine residents, and provide preliminary data supporting the efficacy of this approach.

## Program description

Participants included the intern (i.e. postgraduate year 1, PGY-1) class at a large, urban, tertiary-care hospital. The PGY-1 class consisted of 16 preliminary interns and 27 categorical interns, for a total of 43 participants. During any given module, 16 to 18 interns on the general medical floor participated in the teaching sessions and program evaluation. Week one of the seminar included interns, as well as PGY-2 and PGY-3 residents.

### Program structure

The chief’s seminar curriculum consisted of eleven, four-week long modules. The module topics were: dyspnea, chest pain, fever, abdominal pain, shock, altered mental status (AMS), syncope, jaundice, dizziness, anemia, and acute kidney injury (AKI). Each four-week long module consisted of four, one-hour weekly sessions. The chest pain and AMS modules were exceptions, having had only three sessions because of scheduling conflicts. Every module began with a competitive session during Week 1. Weeks 2 through 4 were composed of small-group discussions examining module subtopics in greater depth.

### Program content

**Figure 1.**
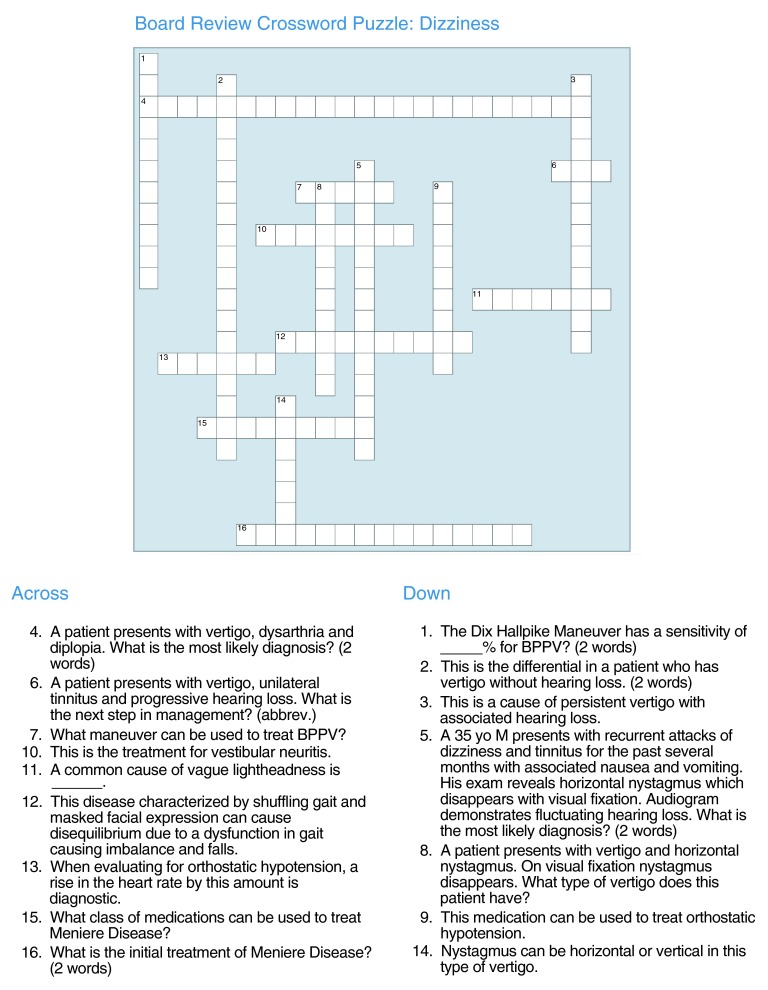
Crossword puzzle for dizziness module. This is an example of a crossword puzzle from the chief seminar module on dizziness ^16^. The questions were targeting high-yield board review topics. Crossword-generating software is readily available via many different websites. We used a free, downloadable program from the following website: http://www.eclipsecrossword.com.

*Week 1*: The first week of the seminar began with a crossword competition that served as an important tool for engaging and motivating residents. All general medical floor interns and residents were included in this competition. The participating house-staff were divided into approximately 6 groups of 4. Each member of the team was given a module-specific crossword puzzle covering all aspects of the chief complaint ( Figure 1). The crossword puzzle was created by the chief residents using a free, downloadable program ( http://www.eclipsecrossword.com). Each month a new crossword puzzle was created, with each puzzle taking 1 to 2 hours to prepare. During this hour-long session, team members worked cooperatively while competing with other teams to complete the crossword puzzle. The team that completed the greatest number of crossword puzzle questions, in the shortest amount of time, won the crossword competition for that module. At the end of the session, all of the answers were reviewed with the house-staff. In addition to the motivation garnered inherently by the competition, teams also competed for $40 worth of gift cards. A photograph of the winning team was distributed via email to the Department of Medicine.

**Figure 2.**
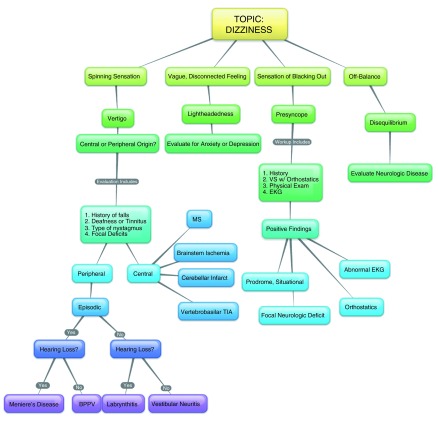
Concept map for dizziness module. This is an example of a completed concept map from the chief seminar module on dizziness ^16^. The concept map starts at the top and progresses downward as more information is collected via intern participation and chief resident guidance. Many online concept mapping programs are available. For this seminar, we used the following website: https://bubbl.us/.

*Weeks 2–4*: The content of the subtopic weeks varied depending on the module, but the same techniques were employed. The emphasis during these sessions was on the generation of a differential diagnosis based on a clinical reasoning algorithm. By using a problem-based approach (i.e. dizziness, not vertigo), the course created a real-life clinical scenario. Prior to the session, a practical clinical reasoning algorithm was created by the chief residents, with each session taking 2 to 3 hours to prepare. At the start of the session, the interns were separated into two, chief resident-led groups. Each subtopic session (three per module) was initiated by drawing the full concept map ( Figure 2). The concept map began with the module title (e.g. dizziness), and then a broad differential diagnosis was determined using an evidence-based clinical algorithm. The algorithm used information gathered from multiple sources, including the history, physical exam, laboratory data, and imaging. For the dizziness module, the initial differential diagnosis was narrowed by using historical questions, then physical exam findings, and lastly by asking targeted questions. Once the concept map was fully developed, a more detailed discussion of one diagnostic pathway (e.g. vertigo) commenced. For each subtopic session, a different diagnostic pathway was discussed in detail. By the end of the module, the intern had an understanding of each diagnosis, as well as an understanding of how the final, targeted diagnosis was derived from the initial, broad differential. The concept map could be handwritten or created via an internet-based program ( https://bubbl.us/).

## Methods

To evaluate the efficacy of our didactic curriculum, we collected pre- and post-course five-question multiple choice tests for each of our modules. Informed consent forms were distributed to all participants and IRB approval for exemption was obtained from Lenox Hill Hospital, North-Shore LIJ (IRB#: 13-045A). Prior to beginning a module, each intern received a unique identifier. This number was used to link the pre- and post-test for each intern participating in the module. The pre-test was given prior to the week 1 crossword competition. The post-test was given after the week 4 teaching session. On completion of a module, pre- and post-test data were entered into a secure, anonymous database according to each unique identifier. Using SPSS Version 20 (IBM SPSS, Chicago IL), data for each module were analyzed via the Wilcoxon Signed-Rank Test. The pre-test and post-test were identical, in order to control for inter-test variability.

**Figure 3.**
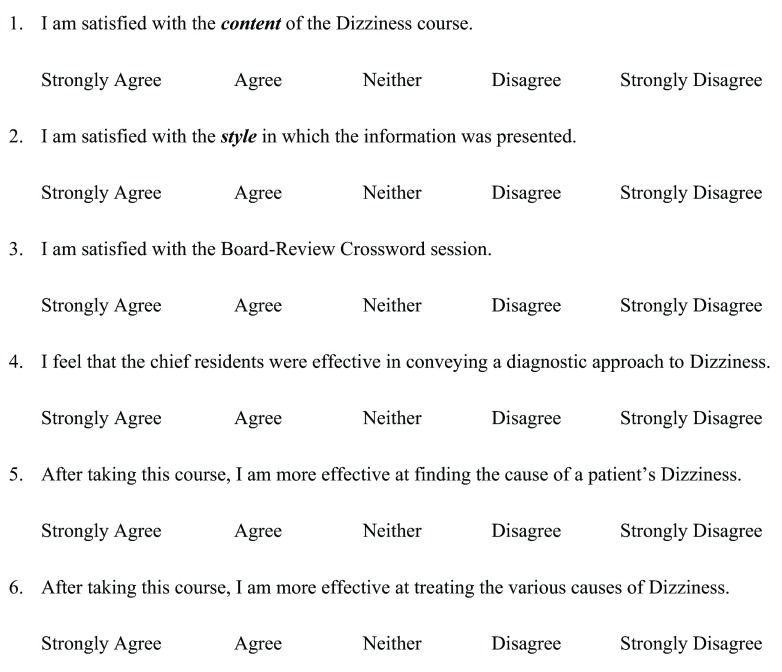
Evaluation form for dizziness module. This is the satisfaction survey evaluation form we used for the dizziness module. We evaluated 6 parameters that were aimed at gaining an overall impression of intern satisfaction with each module.

A six-item resident satisfaction survey was also distributed for each module ( Figure 3). This survey was completely anonymous, and the data collected were descriptive in nature. Survey questions focused on resident satisfaction with the content and style of the module, as well as the perceived effectiveness of the crossword puzzle and small group sessions.

## Results

### Preliminary efficacy results

**Figure 4.**
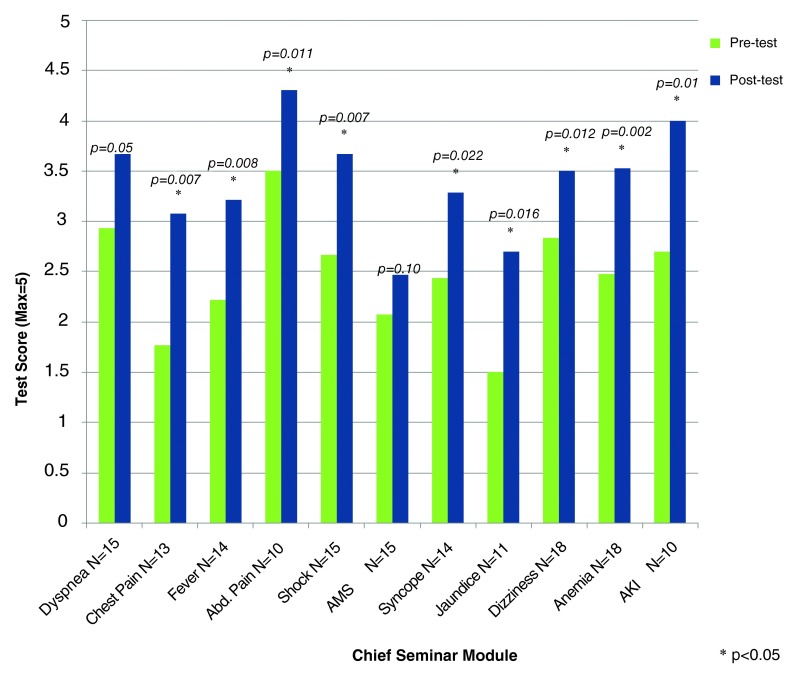
Pre- and post-test scores by module. This figure evaluates the change in score from pre-test to post-test for each of the chief seminar modules. The x-axis lists each module with the number of participants who took both the pre- and post-test. The y-axis has the average participant score (0 to 5). Each module was evaluated for a significant change between pre- and post-test and the p-value can be found at the top of each module’s bar chart.

Efficacy results were obtained for all eleven modules ( Figure 4). Results showed a statistically significant increase from pre-test to post-test score for 9 of the 11 completed modules. Chest pain, fever, abdominal pain, shock, syncope, jaundice, dizziness, anemia, and AKI achieved statistical significance, while the first module, dyspnea, had a trend towards statistical significance. Additionally, the AMS module had, to a lesser degree, a trend towards significance.

### Satisfaction survey results

**Figure 5.**
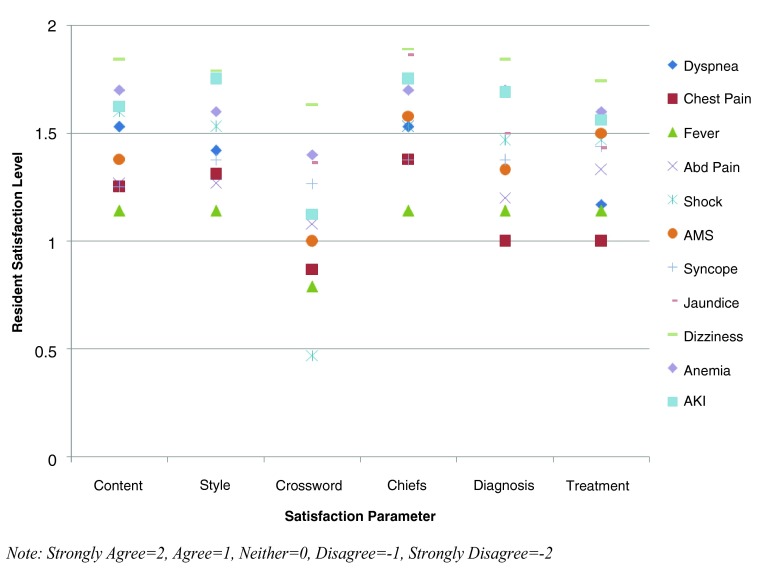
Resident satisfaction survey results by parameter and module. This cluster chart displays the satisfaction survey results for each satisfaction parameter by module. The x-axis lists each survey question and the y-axis lists the level of resident satisfaction, with “2” representing strong satisfaction and “-2” representing strong dissatisfaction.

Intern satisfaction results were obtained for all eleven modules ( Figure 5). Survey results were aggregated by survey parameter for each module. Each response was given a numeric code: Strongly Agree (2 points), Agree (1 point), Neither (0 points), Disagree (-1 point), and Strongly Disagree (-2 points). The aggregate results were then weighted according to the numeric code for each response, and averaged. Of the six survey parameters, five (content, style, chief resident effectiveness, improved ability to diagnose, and improved ability to treat) were at or above the “agree” response for all eleven modules. Only the survey question concerning the crossword puzzle had any responses below “agree”.

Chief Seminar Datasets for Pre-/Post-Testing and Satisfaction Surveys DATASET 1: Pre-test/Post-test Dataset This dataset provides the number of questions correct on the pre-test and the post-test for each participant (code #). Each intern in our residency program was assigned a code number at the outset of the curriculum, which they wrote at the top of their answer sheet. Blank spaces are present because not every intern participated in each module.
DATASET 2: Satisfaction Survey Dataset This dataset provides the results of our satisfaction survey dataset for each survey parameter. The weighted scores can be found at right, with the average score being the final score that was plotted in Figure 5.

## Discussion

The primary goal of our study was to present a reproducible, chief resident-led, teaching curriculum that applies active learning principles to a clinical reasoning seminar for interns. The interns who participated in our study were focused and engaged during our sessions and their positive satisfaction survey results reflect this. We have described our curriculum in detail with the hope that it can be replicated by other residency programs.

The second goal of our study was to present preliminary data evaluating the efficacy of this teaching modality. We have shown a statistically significant increase in test scores for 9 of our 11 modules. For these 9 modules, the interns showed retention of clinical reasoning techniques. Coupled with positive satisfaction surveys, we conclude that this curriculum is both effective and desirable. The remaining two modules, dyspnea and AMS, trended towards, but did not achieve, statistical significance. Dyspnea was our first module, and we were not surprised with the lack of statistical significance. Less clear was why AMS did not reach statistical significance. The broad nature of this topic, combined with one less teaching session, likely contributed to the decreased efficacy outcome for this module. It is also important to note that the number of participants in any of these sessions was low, and if there were more participants, statistical significance would have likely been achieved.

Several limitations of our study have been identified. First, the lack of a comparison group does not allow us to conclude that our teaching curriculum was more effective than a traditional teaching approach. Being a pilot study, the first step was to show that this was an effective teaching method. Subsequent research can build on our preliminary findings by directly comparing this novel curriculum to a traditional, purely lecture-based curriculum. Another limitation was the potential for a practice effect, where the answers to the pre-test are remembered for the post-test. This is most pronounced with a short interval between pre- and post-testing. We did not review any pre-test answers with the participants, and our tests were separated by one month, which was likely sufficient to limit this bias. Another possibility is that the interns searched for the answers to the pre-test before they completed the post-test. We find this unlikely since the post-test scores never approached 100% accuracy. Lastly, our study did not examine long-term retention. This requires the post-test to be repeated at a longer interval, which will be an area of future research.

Despite these, relatively minor, limitations, we have shown that this chief resident-led, evidence-based clinical reasoning approach to graduate medical education is effective and enjoyable for internal medicine residents and we feel it should be applied and adapted throughout residency education.

## Data availability

*figshare:* Chief seminar datasets for pre-/post-testing and satisfaction surveys. Doi: 10.6084/m9.figshare.1157699 ^17^
