# Supplementary material for: Chief’s seminar: turning interns into clinicians
Source: F1000Res. 2014 Sep 8;3:213. [Version 1] doi: 10.12688/f1000research.5221.1 (PMC4184299; doi:10.12688/f1000research.5221.1)
Supplement: Peer review history

# Peer review: Referee response for version 1

**DOI:** 10.5256/f1000research.5567.r6068

**Published:** 2014-09-30

**Authors:** Stewart Babbott

**Affiliations:** 1 Division of General Internal Medicine, University of Kansas Medical Center, Kansas City, USA

Internship is a time of rapid professional and personal growth. Instruction which meets the practical needs of the learners and delivered in ways concordant with the practice environment can be more likely to viewed as successful. This manuscript describes a year long program (11 modules of 4 weeks each) developed for interns to address common clinical situations and diagnoses.

Overall, this was a well thought out and delivered program. The authors sought ways to engage the learners including basing the instruction on active learning techniques.

Title: Turning interns into clinicians is the goal, but the program only brings the interns part of the way to that goal. The title may overstate the scope of the program; however, the authors note this was a pilot program.

Abstract: Provides an adequate summary.

Study design, methods and analysis are appropriate. The goals of the study as stated were to deveop a reproducable chief led program that applied active learning principles to a clinical reasoning seminar (done by survey). A secondary goal was to present preliminary data to evaluate efficacy (done by pre-post scores).

Discussion and conclusions. The authors appropriately discuss the findings, limitations and conclusions; the curriculum was well received and post test scores improved for most of the modules.

Replication: the authors have supplied their surveys, and example of the crossword, the topics and the format within the residency program to allow for replication of the process of the program.

Comment: to follow this study on to further assess the process of turning interns into clinicians, it would be of interest to assess their clinical performance. One approach could be a review of their patients (inpatient and outpatient) for diagnoses addressed and patient outcomes. Another approach is development of an Entrustable Professional Activity around these key areas, and to assess housestaff performance in the relevant venue (eg: shock in the ICU or jaundice in the hospital). I liked the use of the concept map that the learners developed for each module: one way to potentially assess that is to select cases for case conference or morning report, had have the interns use their concept map in the discussion of that case.

I have read this submission. I believe that I have an appropriate level of expertise to confirm that it is of an acceptable scientific standard.

# Peer review: Referee response for version 1

**DOI:** 10.5256/f1000research.5567.r6066

**Published:** 2014-09-25

**Authors:** Janet Wale

**Affiliations:** 1 Convenor, Cochrane Collaboration Consumer network, Brunswick, 3056, Australia

This is a well-written informative and easily understood report on an observational study of a pilot educational program for teaching 1st-year interns, by chief residents. It shows a number of positive results.

It would be interesting to also get feedback from the chief residents for each module; and information on ‘what they would change’ (if anything), particularly as they develop the materials according to a framework.

Some of the limitations of the pilot are clearly identified, which is very useful.

Background, line 11: a word is missing – should read ‘..knowledge base of an attending clinician, while…’

I have read this submission. I believe that I have an appropriate level of expertise to confirm that it is of an acceptable scientific standard.
